# Seatrout (*Salmo trutta*) is a natural host for *Parvicapsula pseudobranchicola* (Myxozoa, Myxosporea), an important pathogen of farmed Atlantic salmon (*Salmo salar*)

**DOI:** 10.1186/s13071-015-0828-z

**Published:** 2015-04-10

**Authors:** Haakon Hansen, Trygve T Poppe, Turhan Markussen, Egil Karlsbakk

**Affiliations:** Norwegian Veterinary Institute, P.O. Box 750 Sentrum, N-0106 Oslo, Norway; Norwegian University of Life Sciences, School of Veterinary Medicine, P.O. Box 5003, N-1432 Ås, Norway; Institute of Marine Research, P.O. Box 1870, 5817 Nordnes Bergen, Norway

**Keywords:** Natural host, Life-cycle, Salmonids, Marine Parasites, Parvicapsulidae, Norway

## Abstract

**Background:**

*Parvicapsula pseudobranchicola* (Myxozoa) causes widespread infections in farmed Atlantic salmon in northern Norway. Heavily infected salmon become runts, probably due to vision impairment or blindness. The salmon are likely infected by waterborne actinospores, released by an alternating annelid host, but the life cycle of *P. pseudobranchicola* is unknown. Seatrout and Arctic charr have been considered possible hosts for the parasite, but firm evidence has been lacking.

**Findings:**

We show for the first time the presence of mature spores of *P. pseudobranchicola* in seatrout. The seatrout were infected with high intensities of *P. pseudobranchicola* in the pseudobranchs in early April. The presence of mature spores in early spring suggests that the fish had been infected late the previous year, a pattern of infection similar to that observed for farmed salmon stocked in autumn. Although heavily infected, the fish did not display any symptoms consistent with parvicapsulosis. The results suggest that the life cycle of *P. pseudobranchicola* is more adapted to seatrout, rather than to Atlantic salmon.

**Conclusions:**

The presence of mature spores of *P. pseudobranchicola* in seatrout confirms that seatrout is a natural host for this myxosporean and this is also the first record of these spores in the pseudobranch of a wild salmonid. Furthermore, wild trout from non-farming areas may become heavily infected with *P. pseudobranchicola*, developing pseudobranch pathology resembling that of farmed Atlantic salmon suffering from parvicapsulosis.

## Findings

### Background

Infections with the myxosporean *Parvicapsula pseudobranchicola* are common in seawater farmed Atlantic salmon (*Salmo salar*) in Norway, especially in the three northernmost counties [1-4]. The known life cycles of parvicapsulids involve a polychaete alternate host [5-7]. However, the life cycle of *P. pseudobranchicola* is unknown.

*Parvicapsula pseudobranchicola* is most commonly diagnosed from the pseudobranch tissue in Atlantic salmon, and disporic trophozoites are produced interlamellary in this tissue [8]. In heavily infected pseudobranchs, there are few intact pseudobranch cells left [9,10]. Foci of infection have also been detected at other sites, but only in heavily infected salmon [9]. In severe infections with clinical parvicapsulosis, the pseudobranch is macroscopically observed to be swollen or papillate, or with a whitish “cheesy” matter sometimes covered with haemorrhages. Occasionally the pseudobranch tissue may be more or less ulcerated. Typical clinical signs include surfacing of a proportion of the fish in the pens; the fish swim disorganised, appear lethargic and may be unresponsive to visual challenge, as if blind. The eyes usually show crescent-shaped haemorrhage and cataract and exophthalmia may also occur. The fish go off feed, and tend to be cachectic and anaemic [9,11]. Parvicapsulosis in farmed Atlantic salmon emerged as a problem in 2002, roughly coinciding with a more widespread autumn stocking of smolts in northern Norway [11]. The discovery of *P. pseudobranchicola* infections in farmed Atlantic salmon suggested that the parasite could be present also in wild salmon, but also led to the assumption that other salmonids might host the parasite. Using real-time PCR, parasite DNA was detected in all three salmonid species present in Norwegian waters; wild Atlantic salmon, sea run Arctic charr (*Salvelinus alpinus*) and seatrout (*Salmo trutta*) [3]. However, the presence of myxosporean DNA in a fish does not provide clear evidence that this particular fish species is a suitable host supporting parasite sporogony. Sporoplasm entry may be unspecific, so unsuitable hosts may be still be PCR positive (see [12]). So far, the presence of mature spores has only been verified in farmed Atlantic salmon, hence the role of Arctic charr and seatrout as potential susceptible hosts is still unclear. Arctic charr are anadromous only in Northern Norway, and can therefore not be involved in the life cycle of the parasite in the southern parts of Norway. Wild Atlantic salmon smolts migrate to the sea during spring, usually spending 1-2 years in oceanic feeding areas before returning to the coast for spawning migration [13]. Since it has been shown from studies on farmed salmon that they develop parvicapsulosis and mature spores of *P. pseudobranchicola* spores in 4-7 months or less [1,4], it seems unlikely that wild salmon play an important role in the natural lifecycle of the parasite. Spores will be produced when the wild salmon is far out at sea and released over great depths, far from potential polychaete hosts. Seatrout, on the other hand, are present in much higher numbers, reside in coastal waters during summer and may overwinter in the estuarine waters [13]. Hence, seatrout may be an important, or even represent the principal vertebrate host for *P. pseudobranchicola*.

To investigate this hypothesis, we collected seatrout in a non-farming area in southern Norway and examined them for infection by *P. pseudobranchicola* and the presence of mature spores.

## Methods

Three seatrout, weighing approximately 1000 g, 600 g and 500 g, were sampled by angling near Rygge, Østfold County (59°18'32.82"N 10°44'31.82"E) on April 2^nd^ 2014. The heads of the fish were collected and kept on ice for 12 hours prior to examination. The pseudobranchs were dissected out and squash preparations made from the pseudobranch. Each squash preparation was subsequently examined for the presence of spores under 400X-1000X magnification in a light microscope. The remaining pseudobranch tissue was cut in half. Half the tissue was fixed in 10% neutral phosphate buffered formalin (12 h) for histological examination. These samples were then transferred to 70% ethanol, embedded in paraffin wax and sectioned (3-5 μm) for histology and *in situ* hybridization. For histology, sections were stained with haematoxylin and eosin (HE) or May-Grünwald Giemsa. *In situ* hybridization was performed as previously described [10]. Images were captured using a Leica DM5000B microscope equipped with a Nikon DXM 1200 digital camera. The other half of the pseudobranchs were stored in 96% ethanol for molecular analyses. A pseudobranch from Atlantic salmon from a fish farm in Northern Norway experiencing parvicapsulosis was used as positive control in the molecular analyses.

DNA was extracted from pseudobranch tissue using the DNeasy Blood and Tissue kit on a QIAcube robot (Qiagen). The DNA extracts were then analysed for the presence of *P. pseudobranchicola* by real-time-PCR [3] and by conventional PCR amplifying 900bp of ribosomal 18S using the primer pair 3LinF and Myxgen4R [14]. PCR products from the latter assay were treated with ExoSAP-IT to remove unincorporated dNTPs and primers, following manufacturer instructions (Affymetrix). Sequencing of a 900 nucleotide 18S rDNA fragment was performed using both amplification primers and the BigDye®Terminator v3.1 Cycle Sequencing Kit. The resulting sequence data were proof-read and assembled with the Vector NTI 11 software (ver. 11.5) (Invitrogen) and subjected to a BLASTn search in GenBank.

## Results

**Figure 1:**
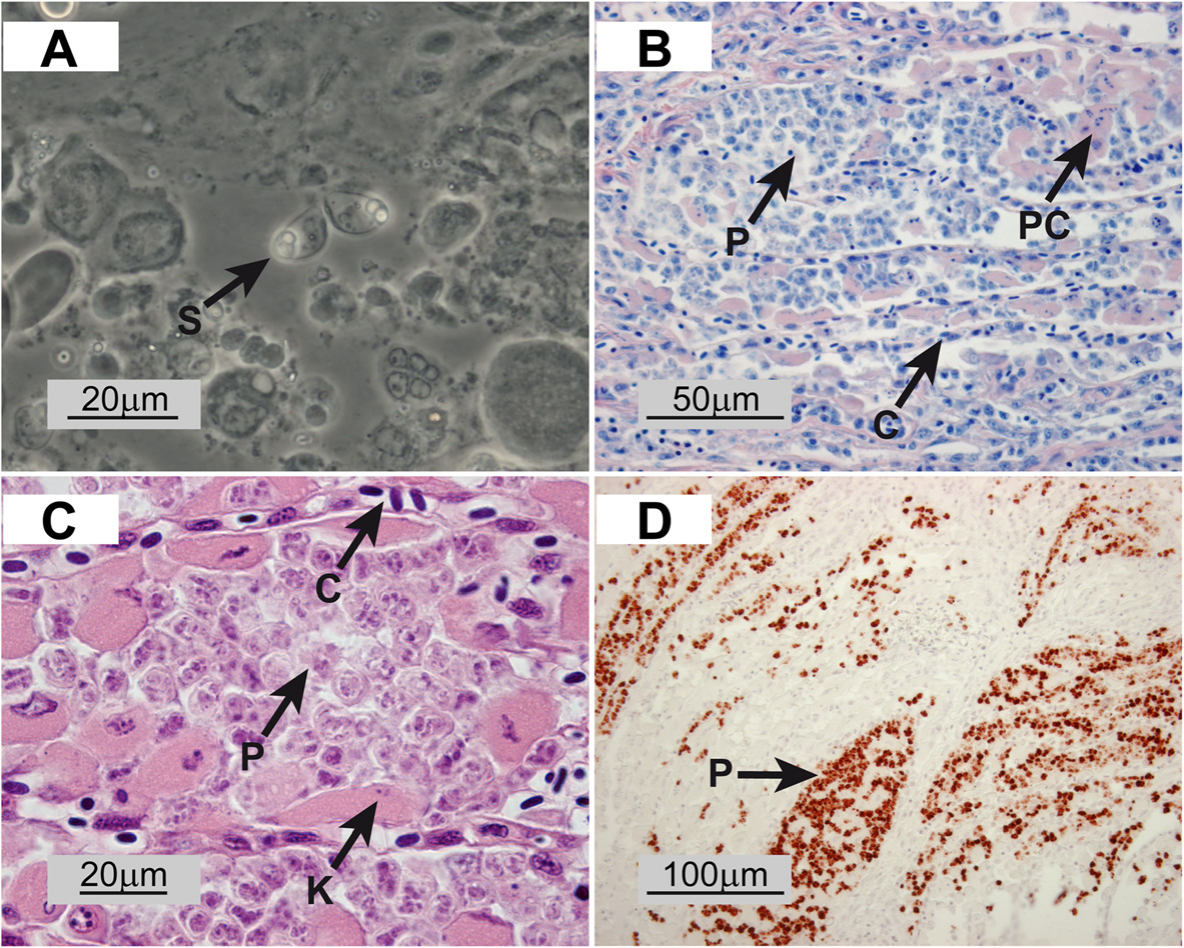
***Parvicapsula pseudobranchicola*** **in pseudobranchs from sea trout (** ***Salmo trutta*** **). A)** Squash preparation showing mature spores (S). **B)** Histological section stained with May-Grünwald Giemsa showing heavily infected pseudobranch tissue. Proliferating spores (P) are clearly observed with few intact pseudobranch cells (PC) left. C = capillaries. **C)** Histological section stained with Haematoxylin-Eosin (HE) showing heavily infected pseudobranch region at higher magnification. Pseudobranch cells (PC) are observed, but the majority are either destroyed or nuclei show karyorheksis (K). C = capillaries, P = proliferating spores. **D)** *In situ* hybridisation showing specific staining in areas with proliferating spores (P) surrounded by more intact pseudobranch tissue (not stained).

The seatrout pseudobranchs examined in the present study appeared macroscopically normal. However, microscopic examination of squash preparations revealed a number of mature spores per field (no attempt to quantify the spores was done) and early developmental stages (trophozoites). The morphology and measurements (data not shown) of the most developed spores matched perfectly the description of *P. pseudobranchicola* (Figure 1A).

Although most of the pseudobranch was heavily affected by the infection with resultant disruption of the normal architecture, small, scattered islands of intact tissue with intact pseudobranch cells lining the lamellae could be identified (Figure 1B, C and D). In affected areas interlamellar spaces were filled with different developmental stages of the parasite, sometimes with associated haemorrhage from disrupted lamellar vessels. Remaining pseudobranch cells were pale and hypertrophic, many of them partly necrotic showing different stages of cell death with pyknosis and karyorhexis (Figure 1A and B). Inflammatory reaction was sparse in the interlamellar regions, while a mononuclear cell infiltrate was obvious near the base of the lamellae.

All three fish tested positive by real-time-PCR, producing Ct’s from 12 to 15; the lowest value was obtained from the largest fish. By comparison, the positive control fish originating from an outbreak of parvicapsulosis produced a Ct-value of 16. Sequencing of samples from all three fish confirmed the presence of *P. pseudobranchicola* with 100% sequence identity (GenBank Acc. no. AY308481).

## Discussion

We show for the first time the presence of mature *P. pseudobranchicola* spores in seatrout. This is also the first record of spores from this parasite species in the pseudobranch of a wild salmonid. These observations confirm that seatrout is a host for this myxosporean. The life cycle of the parasite has been a focus of research since it was first described back in 2002 [11]. Since wild salmon leaves coastal waters on their feeding migrations, their role in the life cycle of the parasite is most likely limited. Ongoing studies on returning wild salmon, based on real-time PCR, suggests they contain low levels of parasite DNA with Ct’s averaging 30 (unpublished data). This is much lower than obtained for seatrout in the present study. Although evidence so far has been lacking, seatrout has been suspected to host the parasite. In our study, we have shown that the parasite life cycle involve sea trout, a species with a different ecology than wild Atlantic salmon. This has implications for the search for the final host of *P. pseudobranchicola* and should result in an increased focus on examination of polychaetes from seatrout habitats in estuaries.

The present observations are at variance with the previously perceived natural infection dynamics of the parasite [4]. Spring stocked Atlantic salmon smolts (April-June) in northern Norway may become PCR positive from July and develop spores in September-October, rarely developing clinical parvicapsulosis [4]. This could mimic a natural situation, as wild Atlantic salmon smolts enter the sea during spring. However, a farming situation with stocking of salmon smolts in the autumn often leads to clinical parvicapsulosis and development of spores during winter-spring (February–May). The seatrout examined in early April from the Oslofjord contained mature spores. These fish were relatively large (i.e. not first seawater sojourn), and may have resided in the sea during winter [13]. This suggests that some seatrout in southern Norway become infected during autumn or winter and release mature spores in the spring (April-May). Consequently, the infection dynamics suggested by our observations on wild seatrout resemble that associated with autumn stocked farmed Atlantic salmon. In previous studies on *P. pseudobranchicola* in wild salmonids, using the same real-time PCR assay as in the present study, high Ct values were observed but no myxospores were detected [3]. However, the results presented here verify that this parasite indeed does infect and develop mature spores in seatrout. Furthermore, this shows that wild trout from non-farming areas may become heavily infected with this parasite, developing pseudobranch pathology resembling that of farmed Atlantic salmon suffering from parvicapsulosis. The examined trout were apparently healthy judged by the fact that they were caught on angling equipment and no pseudobranch lesions were macroscopically evident. In contrast to this, a significant portion of the pseudobranch cells were clearly destroyed. The effect an infection with this myxosporean has on salmonids will depend on the function these principal cells have in this organ, which is presently unknown (see [15]). However, typical clinical signs of parvicapsulosis in farmed Atlantic salmon suggest impaired vision or blindness. Since the ocular blood supply is provided exclusively through the pseudobranchs, the infection may affect retinal oxygenation. In the examined seatrout, no vascular lesions could be found, and only the pseudobranch cells were found to be affected. Inflammation was mainly restricted to the areas close to the filaments. Possibly, inflammation is triggered at a later stage since the formation of exudative lesions may be necessary for spore release to the environment. A seasonal study of *P. pseudobranchicola* infection and development in a natural host, the seatrout, would provide valuable information on this economically important and enigmatic parasite.
